# Integrating pathomics and deep learning for subtyping uveal melanoma: identifying high-risk immune infiltration profiles

**DOI:** 10.3389/fimmu.2025.1585097

**Published:** 2025-07-09

**Authors:** Qi Wan, Ran Wei, Hongbo Yin, Jing Tang, Ying-ping Deng, Ke Ma

**Affiliations:** Department of Ophthalmology, West China Hospital of Sichuan University, Chengdu, Sichuan, China

**Keywords:** unsupervised learning, uveal melanoma, immune infiltration, pathomics features, deep learning

## Abstract

**Purpose:**

Uveal melanoma (UVM) is the most common primary intraocular malignancy in adults, characterized by high mortality despite its relatively low incidence. This study aimed to utilize unsupervised learning techniques to identify a high immune infiltration subtype of UVM and improve patient stratification based on mortality risk.

**Methods:**

A total of 70 hematoxylin and eosin (H&E) stained whole-slide images (WSIs) of UVM were collected from the Genomic Data Commons (GDC) data portal, along with genomic and clinical data. An additional validation cohort of 68 UVM patients from West China Hospital was included. Pathomic features were extracted using CellProfiler software, and deep learning models were constructed for classification and survival prediction. Unsupervised clustering was performed to identify critical regions for prognosis prediction and patient classification. The relationship between histopathological features and genomics was explored.

**Results:**

The study achieved accurate prediction and classification of UVM patients using deep learning models and machine learning techniques. A high immune infiltration subtype of UVM was identified, which showed prognostic relevance. Unsupervised clustering categorized UVM patients into three distinct subgroups. The developed deep learning model based on the Inception-V3 architecture demonstrated promising results in survival prediction.

**Conclusion:**

This study demonstrates the potential of unsupervised learning and deep learning techniques in identifying a high immune infiltration subtype of UVM and improving patient stratification based on mortality risk. This research contributes to the field of computational pathology and highlights the potential of utilizing histopathological images, genomic data, and deep learning models in enhancing the management of UVM patients.

## 1 Background

Uveal melanoma (UVM) is the most common intraocular malignancy in adults and the most frequent type of non-cutaneous melanoma. It is also the primary lethal ocular disease in adults. In the United States, the annual incidence of UVM is reported to be 5.2 cases per million (per year) (1, 2). Based on the cytological morphology of tumor tissues, three subtypes of UVM can be distinguished: spindle, epithelioid, and mixed cell types. Epithelioid cell type has the worst prognosis, accounting for approximately 3-5% of all UVM cases, while spindle cell type has the best prognosis, accounting for over 40% of all UVM cases. The remaining 50% of UVM patients belong to the mixed cell type (3). The tumor cells of UVM originate from melanocytes with pigmentation in the uvea, with 90% of tumors originating from the choroid, approximately 6% from the ciliary body, and 4% from the iris (4). Since UVM is a very diverse tumor, chromosomal changes and gene mutations are thought to be the primary factors for it to arise and spread (5). At present, brachytherapy, proton therapy, enucleation, and stereotactic radiation are the primary approaches used to treat UVM. However, patients diagnosed with UM often face a dismal prognosis primarily due to the high likelihood of metastasis, particularly to the liver, which significantly diminishes survival rates. While local recurrence is not directly linked to poor prognosis, it increases the risk of distant metastases, a major cause of mortality in UVM. Additionally, metastatic UVM cells frequently exhibit resistance to existing therapeutic options, including chemotherapy and targeted treatments, further complicating disease management. Over 50% of primary UVM patients eventually develop distant metastases, with the liver being affected in up to 90% of cases. Typically, the median survival period after metastasis is 10–13 months (6). Patients with uveal melanoma who get early identification and surgical therapy for metastatic UVM may have better overall survival (OS) and progression-free survival. Consequently, prompt diagnosis and therapy are useful steps to enhance the clinical outcome of UVM.

The diagnostic results of pathology directly influence the selection of treatment plans and the prediction of prognosis. Currently, the most frequently employed in clinical practice is the hematoxylin and eosin (H&E) staining of histopathological sections, which is simple, cost-effective, and the preferred auxiliary examination for clinicians (7). In addition, numerous clinical organizations are producing more whole-slide images (WSIs) as a result of developments in scanning equipment, imaging methods, and storage devices. AI and deep learning (DL) methods can be used to examine these images (8). AI-assisted detection and automatic categorization of H&E-stained whole slide images, for example, can aid in identifying the main lesion of malignancies that are unknown in origin (9), grade prostate cancer to a standard that is equivalent to that of skilled pathologists (10), more accurate than conventional cancer staging at predicting the prognosis of patients with colorectal cancer (11), and identify breast cancer’s lymph node metastases (12). Furthermore, our previous studies have demonstrated the accurate prediction and classification of UVM patients using deep learning models and machine learning techniques via WSIs, with an accuracy rate of over 90% in predicting patients’ survival prognosis (13).

Although DL frameworks have achieved impressive performance in segmentation and classification tasks, they still require supervision from pathologists, and the annotation process still demands significant resources (14). To address this limitation, we utilized CellProfiler software for the extraction of pathomics in tumor areas. Pathomics is a new research method that enables automated processing and analysis of a large number of pathological images (15). It calculates features such as cell nucleus and cytoplasm morphology, tissue spatial structure, and extracts valuable information to assist in pathological diagnosis and support disease research (16–18). Due to the presence of different disease subtypes and varying degrees of disease progression, there is evident heterogeneity between cell tissues. The application development of pathomics can be used to explore heterogeneity within tumors, diagnose clinical outcomes, and predict treatment responses (19, 20).

Additionally, through unsupervised clustering, we identified critical regions to predict the prognosis and classification of UVM patients. Additionally, we explored the relationship between histopathological features and genomics in an exploratory manner. We believe that mutations in oncogenes and long-term abnormal expression contribute to the pathological process of transitioning from quantitative to qualitative changes in tissue pathology, and our research aims to provide evidence for this process.

## 2 Materials and methods

### 2.1 Data collection

**Figure 1:**
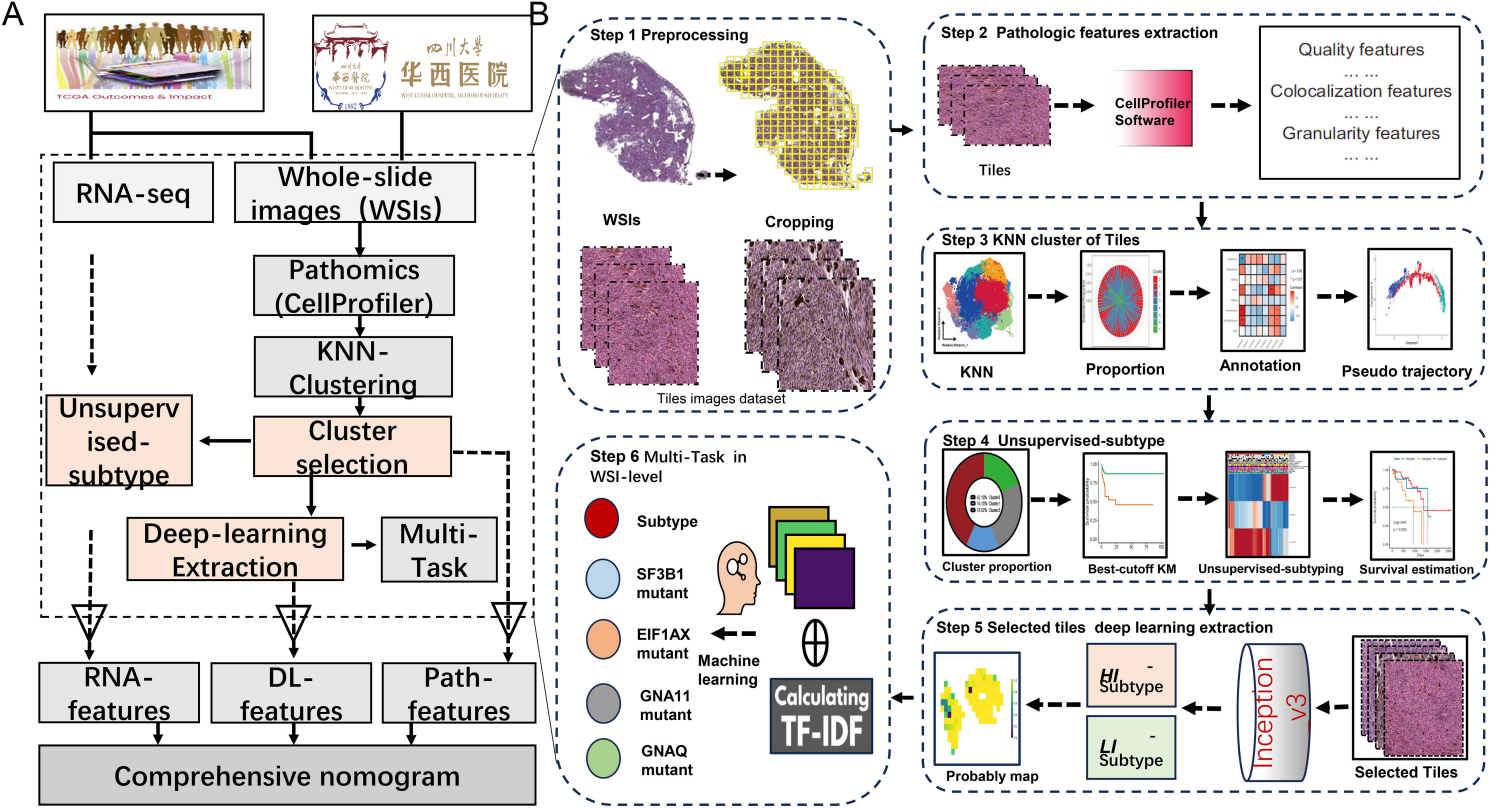
The overall study diagram for the current study. **(A)** The detail flow work for the whole design of study. **(B)** The detail steps of data processing, pathomics analysis and deep-learning network construction.

This study gathered 70 WSIs of uveal melanoma (TCGA-UVM) stained with H&E from the Genomic Data Commons (GDC) data portal, in addition to pertinent genomic information and clinical features such as age, gender, tumor stage, histological type, and metastasis status. Additionally, a validation cohort (WCH-UVM) comprising 68 UVM patients from West China Hospital in Chengdu, China, were included, involving the collection of H&E-stained UVM samples and corresponding clinical data. This study was conducted in accordance with the Declaration of Helsinki. The agreement and written informed consent of WCH-UVM were acquired. The research protocol was reviewed and approved by the Ethics Committee of West China Hospital, Sichuan University (Approval No. 20242000). These WSIs served as the basis for exploring pathomic features and deep-learning features in uveal melanoma patients. The simplified flow diagram for the Study design is illustrated in **Figure 1A**. The work has been reported in line with the REMARK (Reporting recommendations for tumor marker prognostic studies) criteria (21).

To expand the scope of the investigation, additional genomic data from open-access resources (ArrayExpress databases and the Gene Expression Omnibus) were collected. The selection process for appropriate cohorts involved specific criteria: 1) samples originated from human subjects, 2) cohorts included survival data, and 3) cohorts derived from independent studies. Using these criteria, the study incorporated 250 samples from five UVM cohorts (E-MTAB-4097, GSE22138, GSE27831, GSE44295, and GSE84976).

### 2.2 Data processing

First, using Qupath software (v.0.2.3) to achieve accurate identification and delineation of tumor regions within WSIs and ensuring annotation quality, followed by manual review of the WSIs and careful annotation of tumor areas while excluding regions with excessive background or no tissue; subsequently, the WSIs were divided into non-overlapping tiles of 1024x1024 pixels, and pathomics feature extraction as well as deep-learning network construction was based on selecting tiles with a tumor mask overlap of more than 50%; furthermore, the raw RNA-seq data underwent preprocessing steps including probe set conversion, determination of gene expression values, and log2 + 1 transformation for standardization and normalization, thereby providing a standardized foundation for bioinformatic analysis aimed at identifying gene biomarkers.

### 2.3 Pathomics feature extraction

The CellProfiler software is widely used in pathology image analysis, offering automated processing and analysis capabilities for large quantities of pathology images (22, 23). We used CellProfiler (version 4.2.6) to extract quantitative pathomics features of tiles from all tumor regions. Firstly, employ the “UnmixColors” module to separate H&E-stained images into hematoxylin and eosin-stained images. Then, convert the H&E-stained images into grayscale using the “ColorToGray” module, and evaluate image quality features of grayscale H&E, hematoxylin, and eosin images using the “MeasureImageQuality” and “MeasureImageIntensity” modules. Automatically calculate thresholds for each image using the Otsu algorithm to identify tissue foreground from unstained background and extract threshold features. Next, utilize the “MeasureColocalization” module to compute pixel-wise intensity colocalization and correlation between each eosin-stained image and hematoxylin-stained image. Lastly, respectively assess granularity and texture features of each image using the “MeasureGranularity” and “MeasureTexture” modules, outputting a size measurement spectrum for the textures in the image, with the granularity spectrum ranging as specified (15).

### 2.4 Tiles clustering and annotation

Firstly, following previously published protocols and RNA-seq data, we calculated eight indices related to stemness and the microenvironment in Uveal melanoma (24–27). These indices include stemness-related indices (mDNAsi and mRNAsi) and microenvironment-associated indices (DNA methylation of tumor-infiltrating lymphocytes (MeTILs) and the Cancer-associated fibroblasts (CAFs), stromal, immune, tumor purity, and estimate scores). Secondly, we utilized the K-Nearest Neighbor (KNN) algorithm to partition the tiles into eight clusters and employed correlation analysis to annotate the attributes of these clusters. Besides, pseudo-trajectory analysis was performed to compare the similarity of tile clusters between TCGA-UVM and WCH-UVM cohorts.

### 2.5 Unsupervised subtyping

Based on the classification results obtained through the KNN algorithm, we computed the proportions of each cluster in the UVM samples. Kaplan-Meier (K-M) survival analysis was then performed to find clusters that had prognostic importance. Additionally, we employed the “ConsensusClusterPlus(v.1.62.0)” program to categorize UVM patients into three distinct subgroups. This program utilized the unsupervised clustering method “Pam”, with a maximum number of clusters set to maxK=4. The clustering process was performed over 1,000 iterations using Euclidean distance and Ward’s linkage for consistent and reproducible classification.

### 2.6 Ensemble deep learning for multi-task

The tiles in survival-related clusters were selected for deep-learning construction. Two independent datasets were randomly selected from the TCGA-UVM cohort: a train dataset (70%) and a test dataset (30%). The train dataset was utilized to create the model and fine-tune the hyperparameters, while the testing dataset and WCH-UVM dataset were employed for model evaluation. To enhance the diversity and quality of training data, data normalization and augmentation techniques were employed, including horizontal flip, vertical flip, random rotation, etc. Combined with the implementation of a weakly supervised method and subtypes for supervision, an Inception-V3 model was trained over 30 epochs, utilizing the SGD optimizer with a learning rate of 10–2 and L2 regularization with a weight decay of 10-5. Subsequently, a classifier based on the Inception-V3 deep-learning architecture was employed to assign labels to all tiles within each WSI, resulting in the generation of a heatmap depicting the probability prediction for each tile. Considering the large number of tiles within the WSIs, these probability tiles were combined to create a heatmap representing the probability distribution across the entire WSI. The Bag-of-Words (BoW) algorithm was applied to calculate term frequency-inverse document frequency (TF-IDF) features from the probabilities. The TF-IDF features (defined as DL features) were further analyzed to conduct multi-task at the WSI-level via machine learning.

### 2.7 Pathomics and deep-learning -features risk models

Pathomics and DL risk models were constructed to predict patient prognosis using DL-features and clustering proportions. Initially, the candidate whole WSI-level DL features were obtained through lasso regression. These features were then utilized as inputs for the Cox regression model implemented in the “survival” package of R software. The Cox model was employed to calculate the DL risk scores for each patient. Additionally, survival-related clusters were incorporated into another Cox regression model to estimate the pathological risk scores for each patient.

### 2.8 RNA-features construction

On the basis of cluster proportions, UVM patients were divided into three categories. To find differentially expressed genes (DEGs) connected to these subgroups, we used the “limma” approach and set a significance threshold of p < 0.05 and |log2FC| ≥ 2. To further explore the biological mechanisms between different subtypes, we performed Reactome pathway enrichment analysis. Additionally, we used Cox regression to identify prognostic-related DEGs and applied unsupervised clustering to classify UVM patients. The genes positively and negatively correlated with genomic clustering were defined as signature genes p and n, respectively. Next, we utilized the Boruta algorithm to identify important gene subsets within features p and n, followed by performing PCA calculations on these subsets. Finally, we extracted the first principal component (PC1) as the RNA-features. The RNA-features score in UVM samples was calculated as the sum of PC1(p) minus the sum of PC1(n) using the formula: ∑PC1(p) - ∑PC1(n). To assess the predictive capability of the RNA-features, the RNA-features scores of UVM patients were independently examined in six distinct UVM cohorts (TCGA-UVM, E-MTAB-4097, GSE22138, GSE27831, GSE44295, and GSE84976). Each patient was classified as either high-risk or low-risk based on the optimal cutoff score, and the disparity in survival outcomes between the two groups was analyzed using Kaplan-Meier curves and log-rank tests. Additionally, we conducted a meta-analysis to integrate and comprehensively evaluate the risk ratios and survival outcomes across different cohorts.

### 2.9 Statistical analysis

In this study, we utilized Python (v.3.8.0) and R (v.4.2.2) alongside relevant packages to perform various statistical analyses. The proposed deep learning model was implemented using PyTorch, utilizing a GPU (Nvidia GeForce RTX-3080 with 10 GB memory). Machine learning algorithms were executed using the “sklearn” package in Python. K-M and receiver operating characteristic curves (ROCs) were visualized using the “survminer” and “survivalROC” packages, respectively in R. The best cutoff value was decided upon the “survminer” package, while the Pearson test was used to evaluate the association. For comparisons between groups, the Wilcoxon test, and chi-square test were employed. Hazard ratios (HR) and 95% confidence intervals (CI) were computed using Cox regression analysis, with a p-value threshold of less than 0.05 being used to evaluate statistical significance.

## 3 Results

### 3.1 Participants characteristics

This study included two whole slide image (WSI) cohorts and three datasets: the training dataset consisted of 42 consecutive UVM patients from the TCGA-UVM cohort, the testing dataset comprised 42 consecutive UVM patients from the TCGA-UVM cohort, and the validation dataset contained 68 consecutive patients from the WCH-UVM cohort. Statistical analysis of clinical and pathological features revealed no significant differences between the training and testing datasets. However, there were significant differences observed among the three datasets regarding overall survival (OS) time, age, and histological type. The average OS time was 864.12 ± 573.36 days in the training dataset, 704.68 ± 471.53 days in the testing dataset, and 1439.56 ± 973.42 days in the validation dataset. The mean age in the training and testing datasets was 60.40 ± 13.59 years and 64.91 ± 15.11 years, respectively, while the mean age in the validation dataset was 50.81 ± 12.47 years. Furthermore, the proportion of the epithelioid histological type in the three datasets was 16.7%, 18.2%, and 36.8%, respectively (**Table 1**).

**Table 1:** Clinical features of train, test and validate datasets.

Variables	Level	Train	Test	Validate	P	Sig
n		48	22	68		
Overall Survival.time		864.12 ± 573.36	704.68 ± 471.53	1439.56 ± 973.42	<0.001	**
Vital.status		0.33 ± 0.48	0.27 ± 0.46	0.25 ± 0.44	0.634	
MetStatus	Metastatic	17 (35.4)	8 (36.4)	19 (27.9)	0.617	
	Non_metastatic	31 (64.6)	14 (63.6)	49 (72.1)		
Age		60.40 ± 13.59	64.91 ± 15.11	50.81 ± 12.47	<0.001	**
Gender	female	19 (39.6)	10 (45.5)	31 (45.6)	0.797	
	male	29 (60.4)	12 (54.5)	37 (54.4)		
Histological type	epithelioid	8 (16.7)	4 (18.2)	25 (36.8)	0.006	**
	mixed	24 (50.0)	9 (40.9)	13 (19.1)		
	spindle	16 (33.3)	9 (40.9)	30 (44.1)		
SF3B1	mutant	12 (25.0)	4 (18.2)	26 (38.2)	0.123	
	wildtype	36 (75.0)	18 (81.8)	42 (61.8)		
GNAQ	mutant	24 (50.0)	9 (40.9)	32 (47.1)	0.779	
	wildtype	24 (50.0)	13 (59.1)	36 (52.9)		
GNA11	mutant	21 (43.8)	12 (54.5)	24 (35.3)	0.256	
	wildtype	27 (56.2)	10 (45.5)	44 (64.7)		
EIF1AX	mutant	5 (10.4)	3 (13.6)	5 ( 7.4)	0.531	
	wildtype	43 (89.6)	19 (86.4)	63 (92.6)		
Subtype	High_infiltration	15 (31.2)	6 (27.3)	23 (33.8)	0.843	
	Low_infiltration	33 (68.8)	16 (72.7)	45 (66.2)		

### 3.2 Pathomics feature extraction

As shown in step 1 at **Figure 1B**, the WSIs underwent cropping and filtering processes. In the TCGA-UVM cohort, we obtained a total of 30,875 qualified tiles, and in the WCH-UVM cohort, we obtained 18,179 valid tiles. These tiles were then subjected to analysis using CellProfiler, resulting in a final set of 180 quantitative pathomics features available for each tile.

### 3.3 Tiles clustering and annotation

To select a more distinctive subset of tiles from the WSI, we performed clustering on the tiles based on their histopathological features and identified ones with greater discriminatory power. This pipeline consisted of four steps: tiles clustering analysis, calculation of tile category proportions, annotation of tiles, and pseudo trajectory analysis of tiles, as illustrated in step 3 at **Figure 1B**.

**Figure 2:**
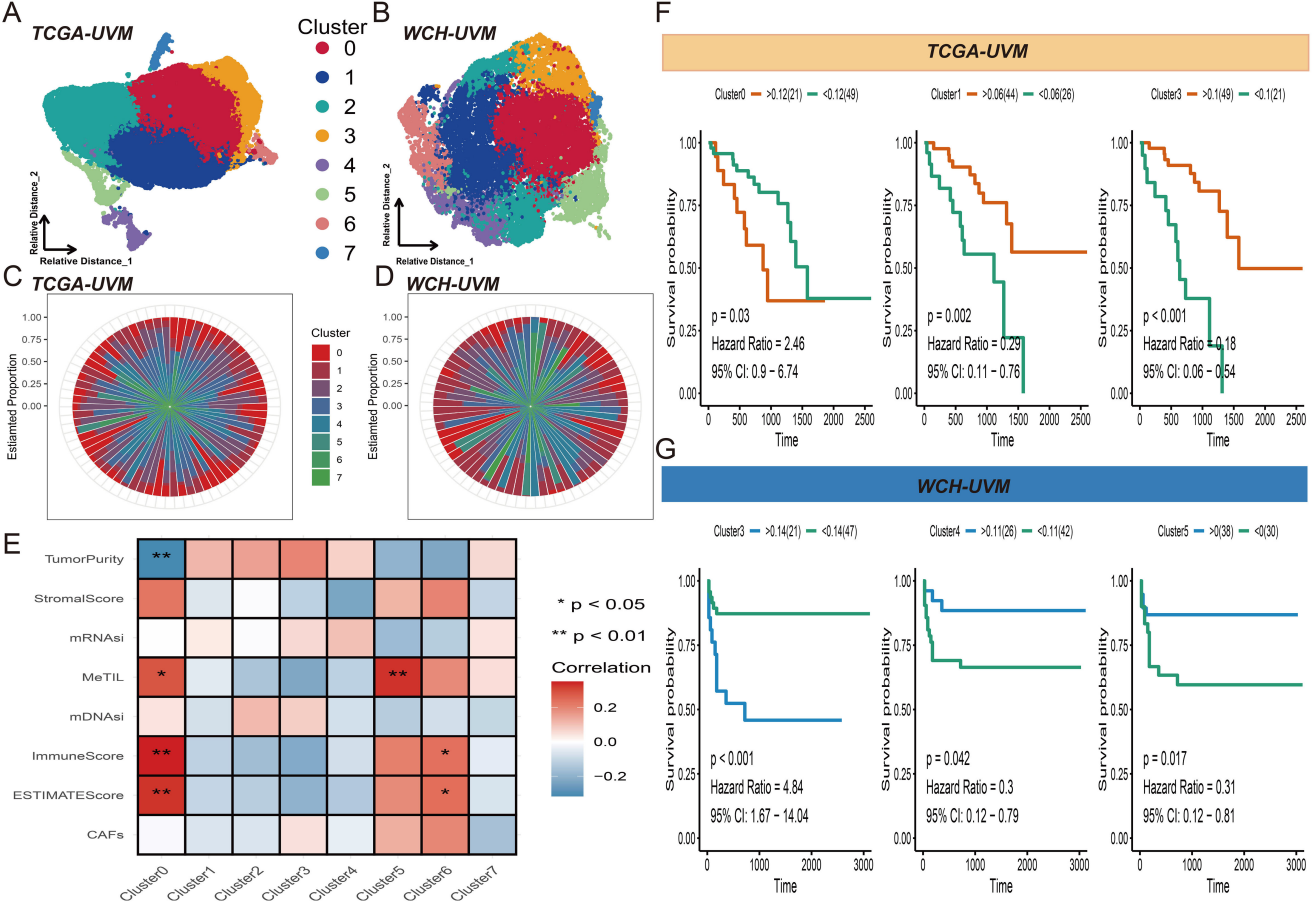
Tiles clustering and annotation. **(A)** Visual representation of the eight clusters of the 30,875 tiles from the TCGA-UVM cohort. **(B)** Visualization of the eight clusters of the18,179 tiles from the WCH-UVM cohort. **(C)** The relative proportions of the eight clusters in the TCGA-UVM cohort. **(D)** The relative proportions of the eight clusters in the WCH-UVM cohort. **(E)** The correlation heatmap of eight clusters and eight indices which included stemness-related indices (mDNAsi and mRNAsi) and microenvironment-associated indices (DNA methylation of tumor-infiltrating lymphocytes (MeTILs) and The Cancer associated fibroblasts (CAFs), stromal, immune, tumor purity, and estimate scores). **(F)** Kaplan–Meier (K-M) curves for survival analysis of Cluster0, Cluster1 and Cluster3 in TCGA-UVM cohort. **(G)** K-M curves of Cluster3, Cluster4 and Cluster5 in WCH-UVM cohort.

Using the KNN algorithm, all 30,875 tiles from the TCGA-UVM cohort were partitioned into eight clusters (**Figure 2A**). Similarly, the 18,179 tiles from the WCH-UVM cohort were also clustered into eight clusters (**Figure 2B**). The proportions of tiles clustering categories in each UVM sample were calculated, and the visualization of the proportions for the eight clusters in the TCGA-UVM cohort and WCH-UVM cohort samples was presented in **Figures 2C, D**, respectively. A correlation analysis heatmap revealed that Cluster0 had a positive correlation with MeTILs, immune scores, and estimate scores, while exhibiting a negative correlation with tumor purity (**Figure 2E**). Furthermore, we incorporated the cluster proportion scores as variables along with patient survival information and performed Kaplan-Meier analysis. In the TCGA-UVM cohort, we found that Cluster0, Cluster1, and Cluster3 were associated with UVM prognosis. Cluster0 had a hazard ratio (HR) of 2.46, indicating a survival risk factor, whereas Cluster1 and Cluster3 had HRs of 0.29 and 0.18, respectively, indicating protective factors for UVM survival (**Figure 2F**). Similarly, in the WCH-UVM cohort, we observed that Cluster3, Cluster4, and Cluster5 were associated with UVM prognosis. Cluster3 had an HR of 4.48, indicating a survival risk factor, while Cluster4 (HR=0.3) and Cluster5 (HR=0.017) were identified as protective factors for UVM survival (**Figure 2G**).

**Figure 3:**
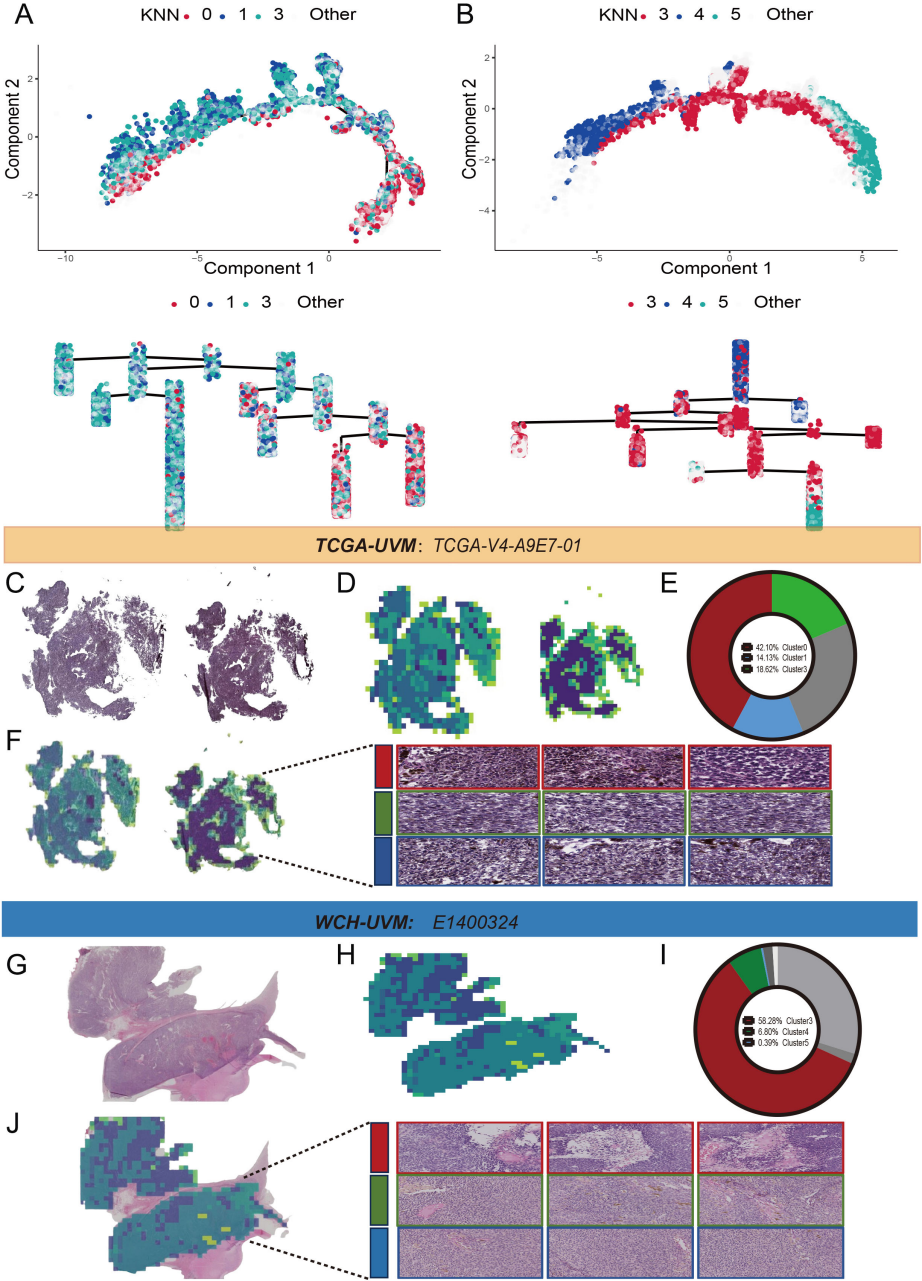
Pseudo trajectory analysis and clusters display. **(A)** The pseudo trajectory and dendrogram tree of Cluster0, Cluster1 and Cluster3 in TCGA-UVM cohort. **(B)** The pseudo trajectory and dendrogram tree of Cluster3, Cluster4 and Cluster5 in WCH-UVM cohort. **(C)** One representative whole-slide image (WSI) in TCGA-UVM cohort. **(D)** Cluster heatmap for WSI-level. **(E)** The relative proportions of the eight clusters in WSI-level. **(F)** The tile clustering and tiles selection for Cluster0, Cluster1 and Cluster3 in representative WSI. **(G)** One example of whole-slide image (WSI) in WCH-UVM cohort. **(H)** Cluster heatmap for WSI-level. **(I)** The relative proportions of the eight clusters in WSI-level. **(J)** The tile clustering and tiles selection for Cluster3, Cluster4 and Cluster5 in example of WSI.

Pseudo trajectory analysis revealed that Cluster0 in the TCGA-UVM cohort (**Figure 3A**) and Cluster 3 in the WCH-UVM cohort (**Figure 3B**) are both distributed on the inner side of the trajectory. On the other hand, Cluster1 and Cluster3 in the TCGA-UVM (**Figure 3A**), as well as Cluster4 and Cluster5 in the WCH-UVM (**Figure 3B**), were distributed on the outer side of the trajectory. Additionally, the dendrogram tree also indicates that these clusters exhibit similar distribution characteristics. To further validate the consistency of the identified categories in both cohorts, we visualized a sample from each dataset separately. **Figure 3C** represents a complete WSI from the TCGA-UVM dataset, and the clustered and stitched heatmap was shown in **Figure 3D**. The pie chart in **Figure 3E** illustrates the relative proportions of Cluster0, Cluster1, and Cluster3. By overlaying the category heatmap with the original image, tile images corresponding to each cluster were identified (**Figure 3F**). Additionally, **Figure 3G** depicted a complete WSI from the WCH-UVM cohort, and the clustered and stitched heatmap was shown in **Figure 3H**. The pie chart in **Figure 3I** illustrates the relative proportions of Cluster3, Cluster4, and Cluster5. Similarly, by overlaying the category heatmap with the original image, tile images corresponding to each cluster were identified (**Figure 3J**).

### 3.4 Unsupervised subtyping

**Figure 4:**
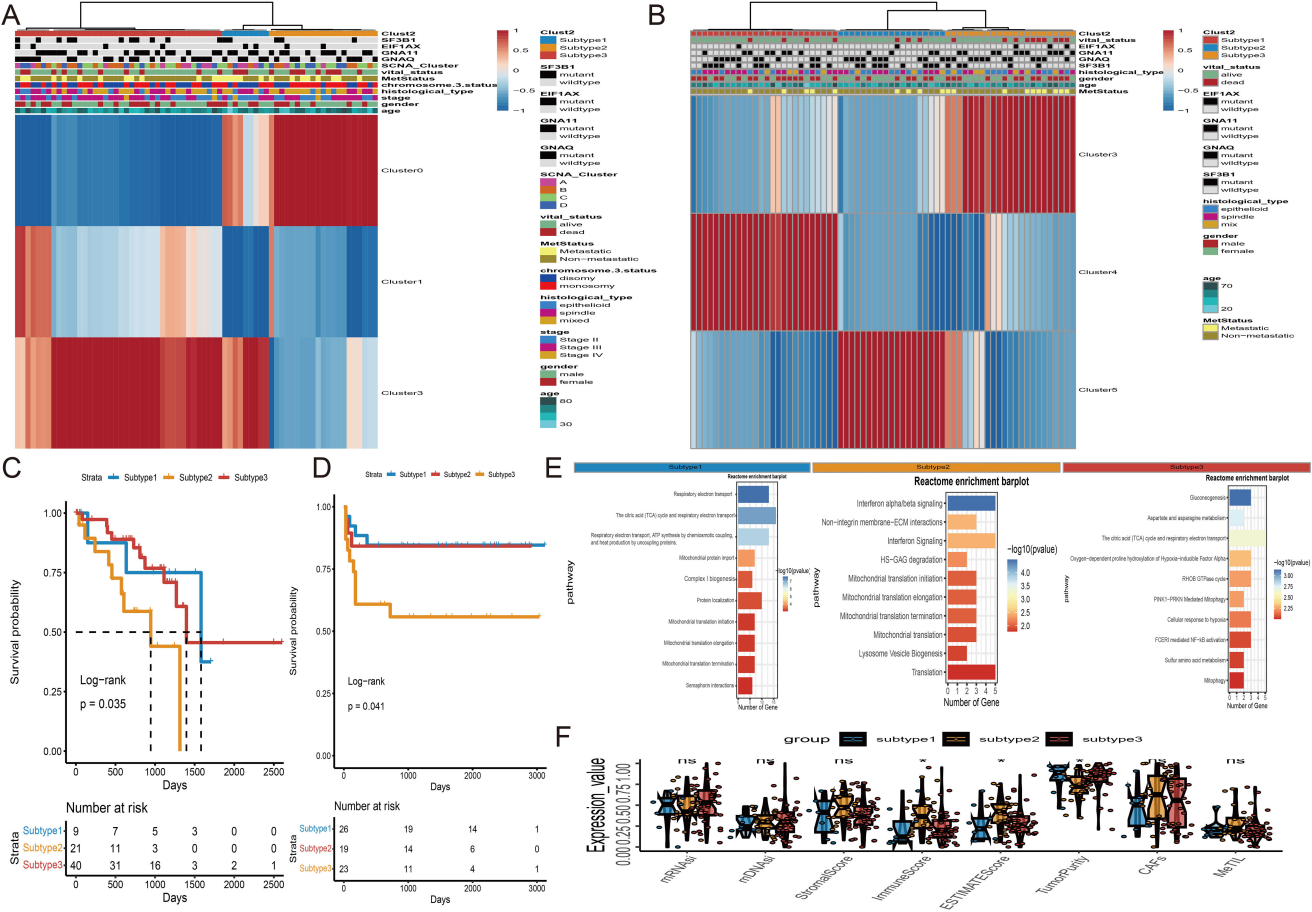
Unsupervised subtyping. **(A)** Subtyping of proportions of the three clusters in TCGA-UVM cohort by an unsupervised clustering method. Proportions of Cluster0, Cluster1 and Cluster3 are shown by rows, and UVM samples are represented by columns. **(B)** Unsupervised Subtyping of proportions of the three clusters in WCH-UVM cohort. Proportions of Cluster3, Cluster4 and Cluster5 are shown by rows, and UVM samples are represented by columns. **(C)** K-M curves for survival analysis of three subgroups of UVM patients in TCGA-UVM cohort. **(D)** K-M curves of three subgroups in WCH-UVM cohort. **(E)** The Reactome pathway enrichment analysis of differentially expressed genes among subgroups. **(F)** Box plots of eight indices in three subgroups. *means p<0.05. mDNAsi and mRNAsi: The stemness-related indices; MeTILs, DNA methylation of tumor-infiltrating lymphocytes; CAFs, The Cancer associated fibroblasts.

Based on survival analysis, we included Cluster0, Cluster1, and Cluster3 from TCGA-UVM, and Cluster3, Cluster4, and Cluster5 from WCH-UVM in the unsupervised clustering analysis. By considering the similarity in cluster proportions, we further separated UVM patients into subgroups. Stable clustering was indicated by a continuous rise in the values of the cumulative distribution function (CDF). Ultimately, through unsupervised clustering (k=3), we identified three stable subtypes. In TCGA-UVM, these subtypes included subtype 1 (9 UVMs), subtype 2 (21 UVMs), and subtype 3 (40 UVMs). Similarly, the WCH-UVM dataset was also divided into three subtypes: subtype 1 (26 UVMs), subtype 2 (19 UVMs), and subtype 3 (23 UVMs). Furthermore, to investigate the connection between subtypes and clinical characteristics, the complete heatmaps of TCGA-UVM and WCH-UVM datasets were displayed in **Figures 4A, B**. K-M survival curve analysis revealed that subtype 2 in TCGA-UVM out of the three subtypes had the poorest prognosis (**Figure 4C**, log-rank p=0.035), while subtype 3 in WCH-UVM had the shortest survival time among the three subtypes (**Figure 4D**, log-rank p=0.041). Reactome pathway enrichment analysis showed that differentially expressed genes in subtype 2 of TCGA-UVM were mainly enriched in immune-related pathways, such as Interferon alpha/beta signaling (**Figure 4E**). In addition, boxplot analysis revealed that immune scores and estimated scores of subtype 2 were significantly higher than those of subtype 1 and subtype 3, while tumor purity was significantly lower than that of subtype 1 and subtype 3 (**Figure 4F**). Therefore, we defined subtype 2 in TCGA-UVM and subtype 3 in WCH-UVM as the high-infiltration subtype, and subtype 1 and subtype 3 in TCGA-UVM, as well as subtype 1 and subtype 2 in WCH-UVM, as the low-infiltration subtype. The different clinical features between high- and low- infiltration subtypes were listed in **Table 2**.

**Table 2:** The different clinical features between high- and low- infiltration subtypes.

Variables	Level	High infiltration	Low infiltration	P	Sig
n		44	94		
Overall Survival.time		800.23 ± 765.41	1272.99 ± 839.84	0.001	**
Vital.status		0.43 ± 0.50	0.21 ± 0.41	0.014	**
MetStatus	Metastatic	18 (40.9)	26 (27.7)	0.174	
	Non_metastatic	26 (59.1)	68 (72.3)		
Age		58.16 ± 13.73	55.56 ± 14.69	0.314	
Gender	female	17 (38.6)	43 (45.7)	0.548	
	male	27 (61.4)	51 (54.3)		
Histological type	epithelioid	13 (29.5)	24 (25.5)	0.099	
	mied	19 (43.2)	27 (28.7)		
	spindle	12 (27.3)	43 (45.7)		
SF3B1	mutant	8 (18.2)	34 (36.2)	0.05	*
	wildtype	36 (81.8)	60 (63.8)		
GNAQ	mutant	21 (47.7)	44 (46.8)	1.000	
	wildtype	23 (52.3)	50 (53.2)		
GNA11	mutant	21 (47.7)	36 (38.3)	0.388	
	wildtype	23 (52.3)	58 (61.7)		
EIF1A	mutant	5 (11.4)	8 ( 8.5)	0.755	
	wildtype	39 (88.6)	86 (91.5)		

### 3.5 Ensemble deep-learning for multi-task

The subtype classifier for diagnosing high-infiltration in uveal melanoma patients underwent rigorous validation using both the TCGA-UVM dataset and the WCH-UVM cohort. The classifier involved two crucial steps: tile-level prediction and WSI-level prediction.

**Figure 5:**
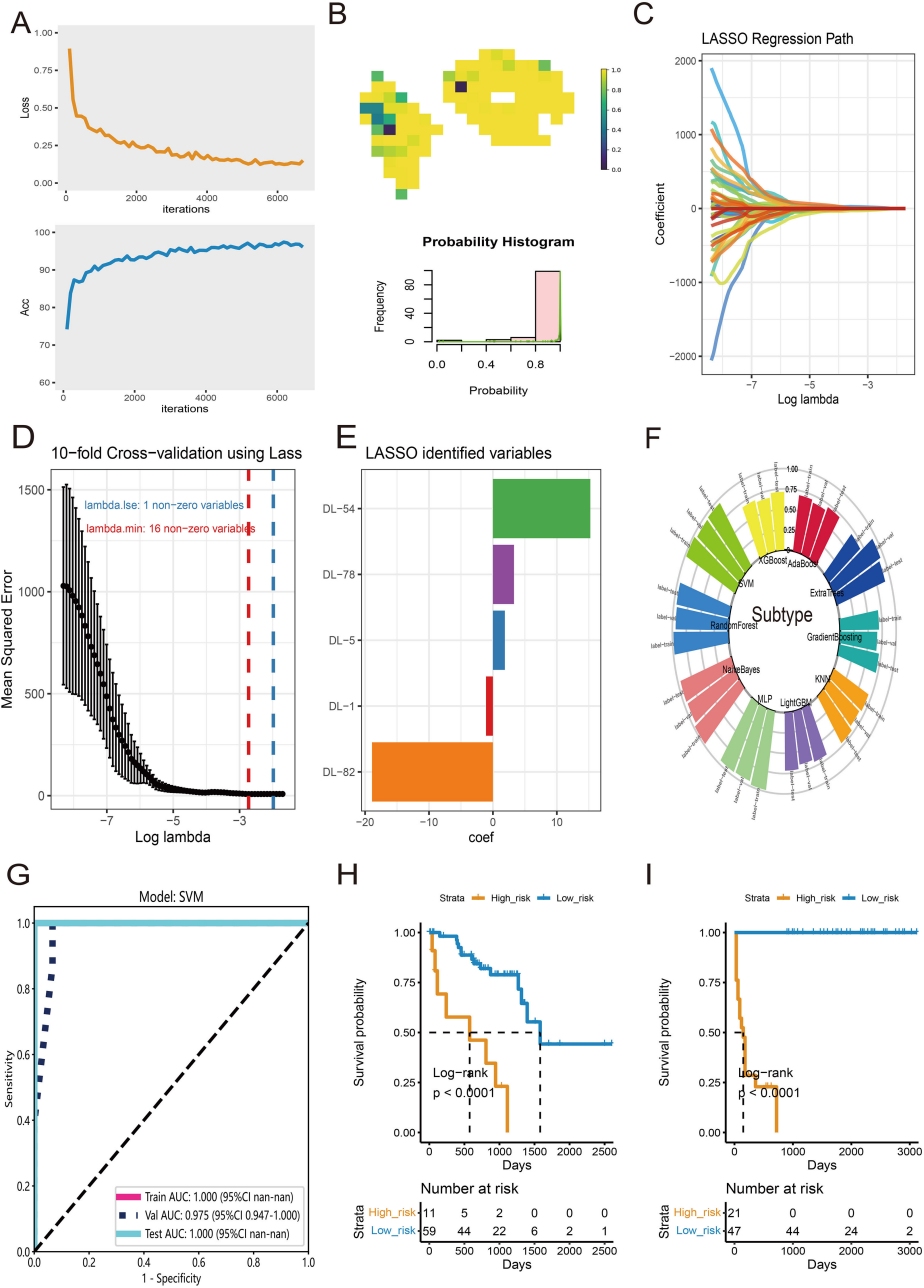
Features selection and diagnosis for high-infiltration subtype at WSI-level. **(A)** The training convergence for Inception-V3: loss curve and accuracy curve. **(B)** An example of probable heatmap and histogram of probability. **(C)** The coefficient profiles of DL features in Lasso regression. **(D)** The distribution of mean squared error with the corresponding λ-logarithm value in 10-fold cross-validation using Lasso regression. **(E)** The selected DL features with coefficients > 0. **(F)** The accuracy distribution of ten machine learning algorithms for classify of subtype. **(G)** The ROC curves of SVM model for prediction of high-infiltration subtype in train, test and validate datasets. **(H)** K-M survival analysis of High- and low-risk groups of UVM patients in TCGA-UVM cohort. **(I)** K-M curves of High- and low-risk groups in WCH-UVM cohort.

For tile-level prediction, 20,689 tiles from survival-related clusters were selected to construct a deep learning model. Subsequently, we randomly split the dataset of 20,689 tiles into training and validation sets at a 1:1 ratio. The performance of the Inception-V3 deep learning model was rigorously evaluated using ROC curves, precision-recall curves, and confusion matrices to ensure a reliable and robust assessment. The specific metrics and detailed performance results of the model are provided in **Supplementary Figure S1**. The convergence of accuracy and loss curves indicated that as the training epoch increased, accuracy approached 100% and loss approached 0% (**Figure 5A**). At the WSI level, multiple probable tiles were combined to create a comprehensive heatmap and corresponding histogram (**Figure 5B**). The entire slide was assessed using the Bag-of-Words (BoW) algorithm, extracting 101 deep learning (DL) features from the histogram of tile probabilities. After adjusting for false discovery rate (FDR) using the Wilcoxon test, 52 DL features showed significant differences with a p-value < 0.05. These features were then utilized in Lasso regression for dimensionality reduction (**Figure 5C**). The optimal lambda value was determined through 10-fold cross-validation (CV) (**Figure 5D**), leading to the identification of five DL features with coefficients > 0 as significant candidate variables (**Figure 5E**). These five features were employed in ten machine learning algorithms, and the Support Vector Machine (SVM) classifier was selected for high infiltration prediction based on the distribution of accuracy results (**Figure 5F**). The ROCs in the train, test, and validation datasets were 1.00, 1.00, and 0.975, respectively (**Figure 5G**). Subsequently, the five DL features were used as inputs for the Cox regression model to calculate risk scores for each patient. High- and low-risk groups of UVM patients were created by using an ideal cutoff value. In the TCGA-UVM dataset (**Figure 5H**, log-rank p < 0.0001) and the WCH-UVM cohort (**Figure 5I**, log-rank p < 0.0001), K-M survival analysis showed a poorer prognosis for high-risk patients.

**Figure 6:**
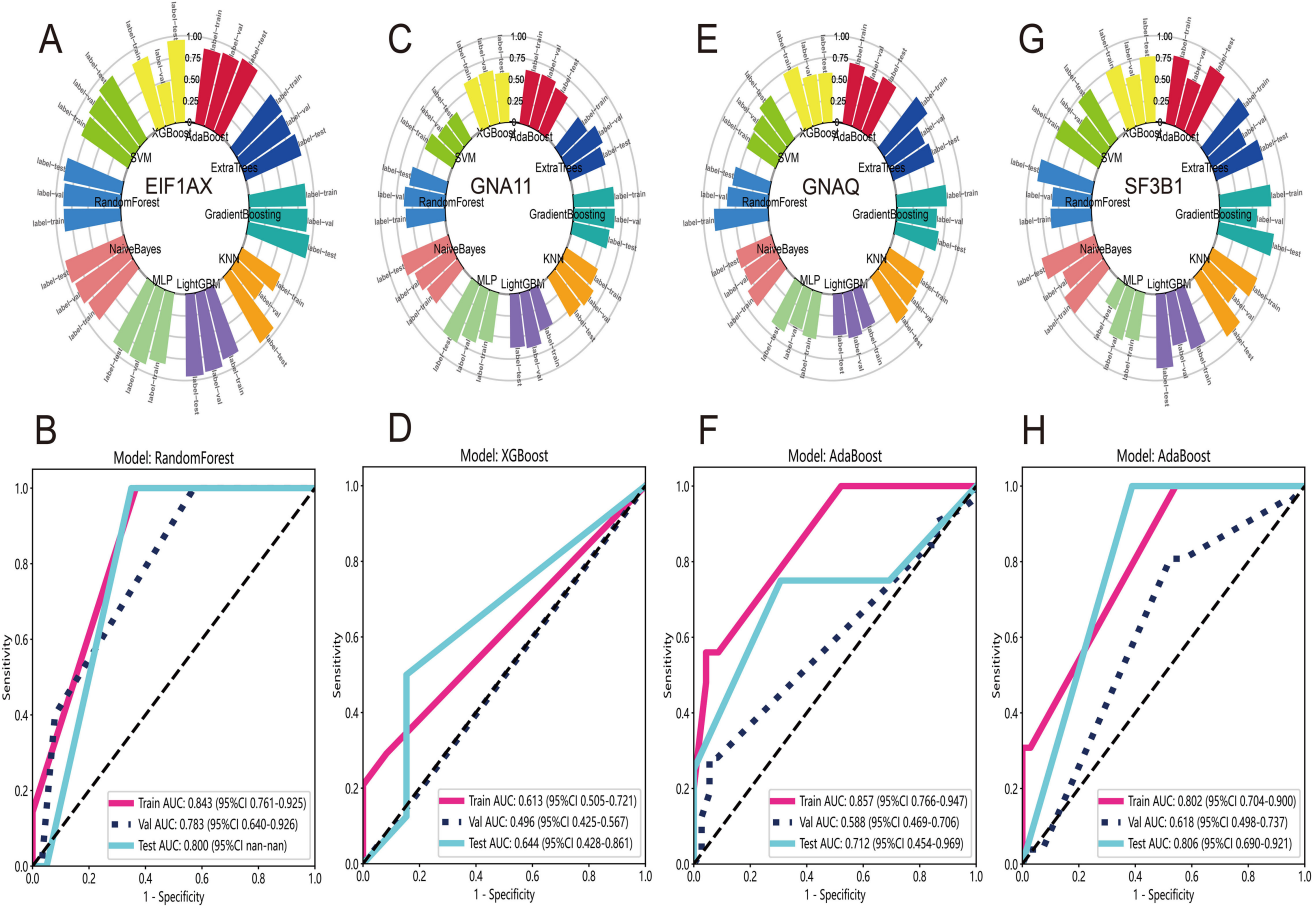
Prediction of gene mutation at WSI-level. **(A)** The accuracy distribution of ten machine learning algorithms for predicting mutation of EIF1AX. **(B)** The ROC curves of RandomForest model for predicting mutation of EIF1AX in train, test and validate datasets. **(C)** The distribution of accuracy for predicting GNA11 mutation. **(D)** The XGBoost model’s ROC curves for GNA11 mutation prediction in train, test, and validate datasets. **(E)** The distribution of accuracy for predicting GNAQ mutation. **(F)** The AdaBoost model’s ROC curves for GNAQ mutation prediction in train, test, and validate datasets. **(G)** The distribution of accuracy for predicting mutation of SF3B1. **(H)** The ROC curves for prediction of SF3B1 mutation via AdaBoost model in train, test, and validate datasets.

In UVM patients, the most common gene mutations include *EIF1AX*, *GNAQ*, *GNA11*, *SF3B1*, and so on. Previous studies have shown that these mutations result in abnormal activation of cellular signaling pathways, promoting the proliferation and metastasis of melanoma cells. Therefore, the use of DL features to predict gene mutations would be beneficial for the treatment and management of UVM patients. Firstly, we employed the Wilcoxon test and FDR adjustment with a significance level of p < 0.05 to select DL features that exhibited differential gene mutations. Subsequently, we utilized ten machine learning algorithms to evaluate the accuracy of the predictions. The distribution of accuracy results indicated that RandomForest had the highest accuracy for predicting *EIF1AX* mutation (**Figure 6A**). The ROCs in the train, test, and validation datasets were 0.843, 0.800, and 0.783, respectively (**Figure 6B**). For the prediction of *GNA11* mutation, XGBoost performed relatively better than other algorithms (**Figure 6C**). The AUCs were 0.613, 0.644, and 0.496 in the train, test, and validation datasets (**Figure 6D**). Regarding the prediction of *GNAQ* (**Figure 6E**) and SF3B1 mutations (**Figure 6G**), AdaBoost outperformed other algorithms. The AUCs of AdaBoost for *GNA11* mutation were 0.857, 0.712, and 0.588 in the train, test, and validation datasets (**Figure 6F**). In predicting *SF3B1* mutation, the AUCs of AdaBoost in the train, test, and validation datasets were 0.802, 0.806, and 0.618, respectively (**Figure 6H**).

### 3.6 RNA-features construction

**Figure 7:**
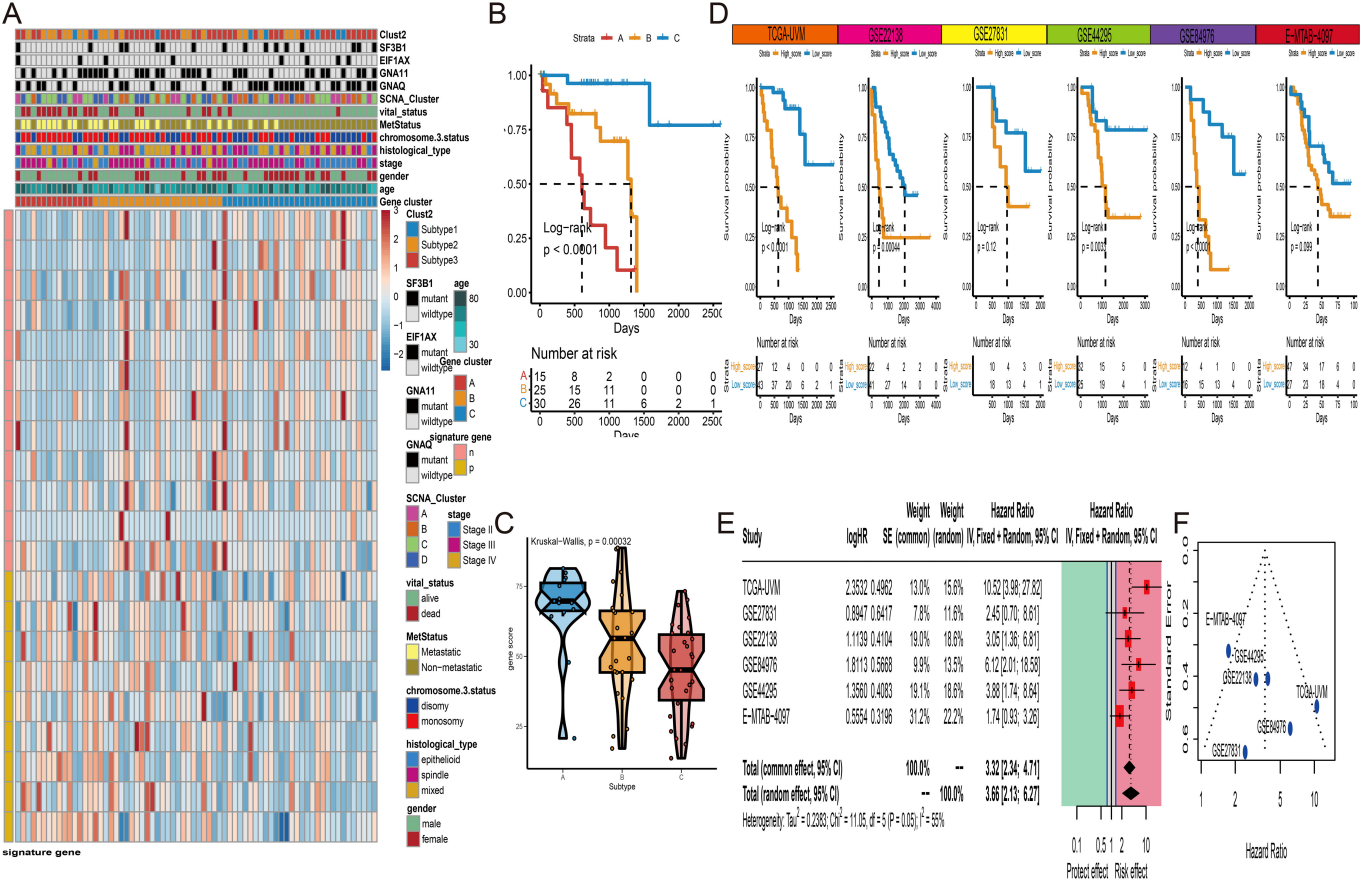
Gene cluster and RNA-features construction. **(A)** Unsupervised clustering of three subtypes (Cluster A to C) based on the expression of differentially expressed genes (DEGs). **(B)** K-M curves of three gene clusters in TCGA-UVM cohort. **(C)** The distribution of RNA-features scores among three gene clusters. **(D)** K-M curves of high- and low-score groups of UVM patients in six independent cohorts (TCGA-UVM GSE22138, GSE27831, GSE44295, GSE84976, and E-MTAB-4097). **(E)** Meta-analysis of hazard ratios for RNA-features scores among six independent UVM cohorts. **(F)** The funnel plot of meta-analysis. * P<0.05; ***P<0.001.

The development of RNA risk features is of significant importance for understanding the prognosis and potential therapeutic strategies of UVM. We utilized the TCGA-UVM cohort as a train set and employed five external validation sets (GSE84976, GSE27831, GSE22138, GSE44295, and E-MTAB-4097) to ensure the robustness of the study results. Firstly, in the TCGA-UVM cohort, we identified 457 differentially expressed genes (DEGs) based on different subgroups. Subsequently, using survival information and univariate Cox analysis, we identified 21 survival-associated DEGs. Based on these 21 genes, UVM samples were classified into three gene-related clusters. The feature p gene set consisted of 12 DEGs positively correlated with the gene clusters, while the feature n comprised 9 DEGs negatively correlated with the gene clusters. A visual heatmap was generated to illustrate the relationship between gene-related clusters and clinical features (**Figure 7A**). The log-rank test revealed that Cluster C exhibited a better survival prognosis (**Figure 7B**). We then used feature n and feature p, respectively, to further identify significant gene sets using the Boruta technique. Ultimately, the *JUP* gene was selected from feature p, and the *UFC1* gene was selected from feature n for PCA calculations. Based on the formula, we obtained RNA-features scores for each UVM patient. The boxplot showed that scores of Cluster C were significantly lower than Cluster A and B (**Figure 7C**). Subsequently, using an optimal cutoff value, UVM patients were divided into high-score and low-score subgroups. The Log-rank test in the K-M curve demonstrated that patients with high scores had a poor overall survival than those with low scores (**Figure 7D**). Finally, a meta-analysis of all cohort results confirmed that RNA-features are an important risk factor influencing UVM survival, with a hazard ratio of 3.66 (**Figure 7E**). The funnel plot displayed a symmetric distribution on both sides of the centerline, indicating a low publication bias in the meta-analysis (**Figure 7F**).

### 3.7 Nomogram construction

**Figure 8:**
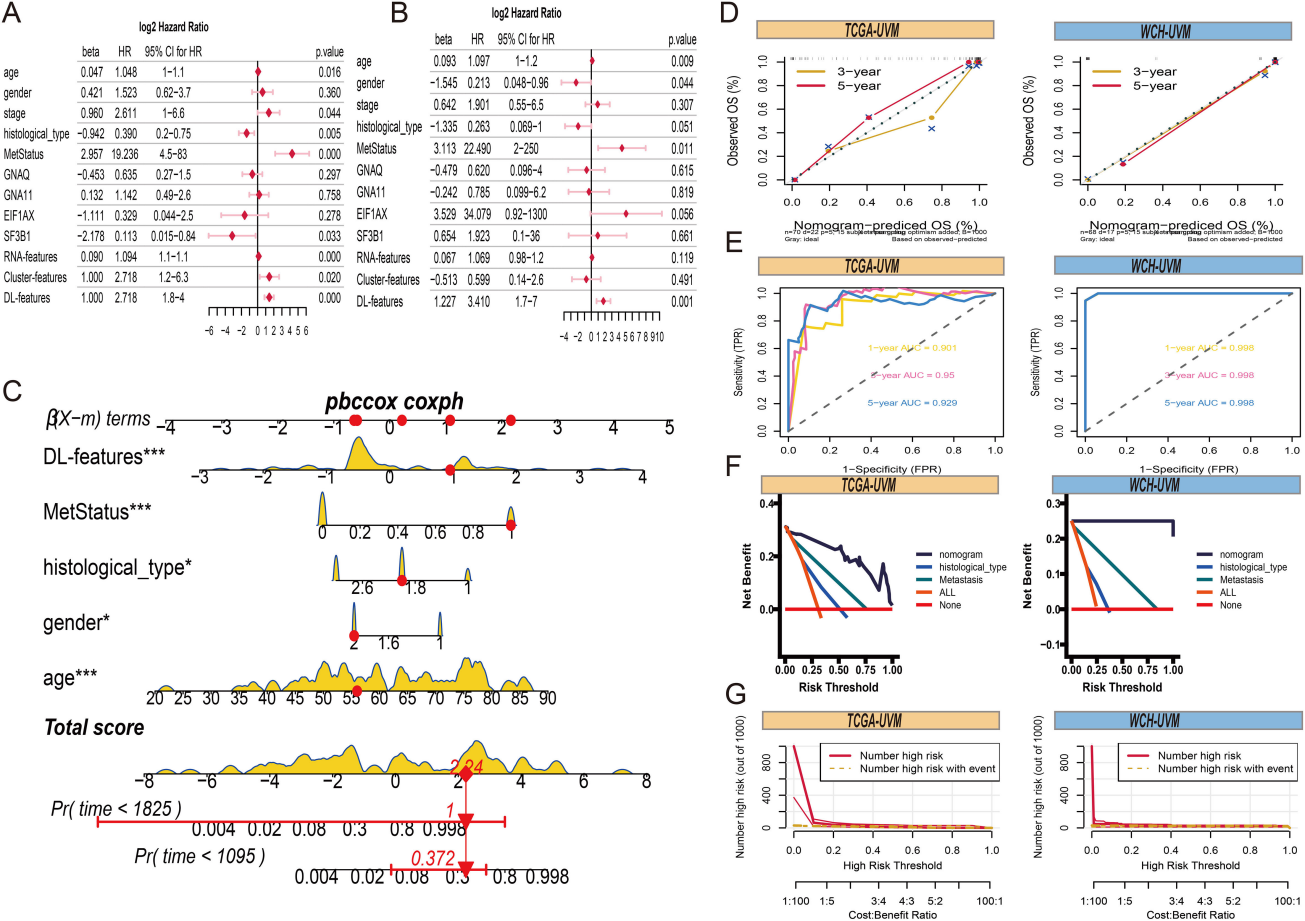
Comprehensive nomogram construction. **(A)** The forest plots present the results of univariate Cox regression analyses for clinical parameters, RNA-features, Cluster-features and DL-features. **(B)** The forest plots of results from multivariate Cox regression of clinical parameters, RNA-features, Cluster-features and DL-features. **(C)** A comprehensive nomogram is used to predict the 3- and 5-year overall survival time for patients in the TCGA-UVM cohort, incorporating DL-features, age, gender, histological type, and metastasis status. **(D)** The 3- and 5-year of calibration curves for overall survival prediction in TCGA-UVM and WCH-UVM cohorts. **(E)** The ROC curves of nomogram model for survival prediction in TCGA-UVM and WCH-UVM cohorts. **(F)** The decision curves of nomogram, histological type, and metastasis for comparison of net benefit. **(G)** The clinical impact curve of the nomogram for risk prediction in TCGA-UVM and WCH-UVM cohorts.

To provide a comprehensive and accurate prognostic prediction method for UVM, we developed a comprehensive nomogram model. First, univariate Cox analysis revealed that DL-features, Cluster-features, RNA-features, age, stage, histological type, metastasis status, and *SF3B1* mutation were correlated with overall survival (OS) (**Figure 8A**). However, in the multivariate Cox analysis, only age, gender, histological type, metastasis status, and DL-features were significantly associated with OS in UVM patients (**Figure 8B**). Therefore, we constructed a comprehensive nomogram model incorporating age, gender, histological type, metastasis status, and DL-feature to estimate the probabilities of 3 years and 5 years OS (**Figure 8C**). In the TCGA-UVM and WCH-UVM cohorts, the 3 years and 5 years nomogram calibration curves showed a significant degree of overlap between the actual and anticipated survival rates, demonstrating good predictive value (**Figure 8D**). Time-dependent ROC curves were used to assess the accuracy of the nomogram, and the AUC values for 1 year, 3 years, and 5 years predictions were all greater than 0.9, indicating good sensitivity and specificity of the nomogram (**Figure 8E**). Additionally, decision curve analysis (DCA) revealed that our nomogram model, which integrates pathomics features with traditional clinical features such as histological type and metastasis status, yielded higher net benefits compared to models relying solely on clinical features (**Figure 8F**). To assess the nomogram’s clinical usefulness, we further generated a clinical impact curve (CIC) using the DCA data. The nomogram’s higher overall net benefit within a broad and useful range of threshold probabilities was intuitively shown by the CIC, which also impacted prediction accuracy and showed the model’s strong predictive value (**Figure 8G**).

## 4 Discussion

Traditional pathological examinations are conducted by experienced pathologists who assess tumor cell characteristics under multiple magnifications. However, pathologists typically do not provide detailed quantitative information for every region of a whole-slide image, and variability in pathological classification and diagnosis can occur due to the heterogeneity of histological subtypes and differences in individual interpretation. Therefore, pathomics can be used as a useful adjunct to more conventional pathological assessments. Our results demonstrate that, without any supervised information, important subregions of each WSI can be identified and objectively quantified through pathomics features. Additionally, by combining genomic information, we can effectively define the cellular biological characteristics of different subregions. Consequently, based on the relative proportions of these three subregions, we successfully distinguished three tumor subtypes (Subtype 1-3) and identified a high-infiltration subtype for UVM patients. We found that UVM patients in the high-infiltration subtype had higher immune scores, estimate scores, and MeTILs compared to the low-infiltration subtype, which correlated with poorer survival outcomes. These observations are consistent with previous research (28, 29). For instance, Narasimhaiah et al (30), have demonstrated that increased infiltration of immune cells, particularly T lymphocytes and macrophages, is linked to metastatic progression in UM and associated with poor prognosis. The eye is an immune-privileged organ, characterized by numerous immunosuppressive elements that prevent robust immune responses. This immune privilege is maintained by mechanisms that hinder the trafficking of activated T cells into tumor tissues and promote T cell exhaustion. As a result, the phenotype of infiltrated immune cells is often altered, converting their anti-tumor functions into pro-tumor roles. Extensive evidence suggests that immune cells linked with tumors in the UVM microenvironment stimulate both immune evasion and immunological repression (31, 32). Tumor-infiltrating lymphocytes (TILs) include CD8+ T cells and CD4+ T cells, for example, are unique independent prognostic markers for UVM patients and play essential roles in tumor recurrence, metastasis, dissemination, and responsiveness to immunotherapy (33–35). In general, our research indicates a potential close relationship between the histomorphology and underlying molecular composition of tumors.

Furthermore, our study is the first to apply integrated deep learning (DL) networks to learn from the entire WSI for diagnosing the immune infiltration subtypes of UVM patients and predicting their common gene mutation information. Our approach has two prominent advantages. First, it analyzes a collection of patches clusters automatically selected from several important subregions associated with prognosis, avoiding any manual annotation. Second, it assigns labels to each patch image through weakly supervised methods and aggregates local features using multiple instance learning to achieve global diagnosis. This approach eliminates the need for manual annotation to describe cancerous regions at the pixel level. Our study demonstrates that the integrated DL network we constructed achieves a significant accuracy rate of over 95% in predicting immune subtypes at the tile level and WSI level. Additionally, our model performs well in predicting *SF3B1* and *EIF1AX* gene mutations. Previous studies have found a close association between *SF3B1* and *EIF1AX* gene mutations and tumor metastasis, with most UVM patients with *SF3B1* mutations eventually developing metastasis (36). However, it is rare for UVM patients carrying only *EIF1AX* gene mutations to experience metastasis (37). Moreover, the Cox survival model constructed using DL features can effectively distinguish high-risk and low-risk groups. Therefore, this model has broad clinical applications, enabling patients to obtain accurate predictions of metastasis and prognosis while receiving pathological diagnoses.

However, in clinical practice, it is insufficient to evaluate the progression and prognosis of UVM solely based on one data type. Therefore, based on the genomic data of different subtypes, differential expression genes (DEGs) were identified among these subtypes. Subsequently, we created a predictive gene signature in the TCGA-UVM cohort based on these DEGs, and we verified the prognostic significance of this signature in many separate datasets. The *JUP* and *UFC1* genes are included in this gene signature. Junction plakoglobin (*JUP*) is an important cell-cell adhesion protein. *JUP* has been identified as a protein with great potential as a biomarker and therapeutic target for UVM (38). Recent studies have found that deregulation of JUP leads to the occurrence and progression of various malignancies (39). Hu et al. found that JUP can regulate the expression of Anterior Gradient 2 (*AGR2*)/*LY6*/*PLAUR* Domain Containing 3 (*LYPD3*) and mediate an immunosuppressive microenvironment in melanoma (40). Additionally, numerous studies have discovered a link between tumor invasion and elevated expression of the long noncoding RNA *UFC1*. Certain cancer cells’ ability to proliferate, migrate, and invade can be inhibited by knocking down *UFC1*, whereas cell cycle arrest and death are encouraged (41–44). Therefore, the gene signature identified through histopathological analysis can serve as a predictive biomarker in future clinical research. Finally, we combined traditional clinical features, histopathological cluster features, DL features, and RNA features for univariate and multivariate Cox regression analysis. We found that DL features, along with age, gender, histological type, and metastasis status, can serve as independent prognostic factors for UVM. Therefore, we integrated these features to construct a comprehensive nomogram model. The model has been demonstrated to have high predictive ability and net benefits in clinical practice, guiding physicians in the rational management of patients.

In summary, our work has several advantages compared to previous studies in computational pathology. Firstly, it addresses several key challenges: (1) It does not require annotation by pathologists but uses histopathological features for unsupervised clustering to identify important subregions within WSIs and perform subtyping analysis of tumor patients. (2) The deep-learning algorithm does not require pixel-level or patch-level annotations because it is trained using simply tumor type as a weak supervisory label. (3) By combining deep learning and multi-omics data, we provide a modern framework for understanding tumor heterogeneity and prognostication in UM. However, despite including samples from 318 patients from different countries and hospitals, future international multicenter and multiethnic datasets are desirable.

In addition, we acknowledge the limitations of our WSI (whole slide imaging) dataset, as the number of slides used in this study was relatively small, and the slide scanners employed were largely uniform. To address these limitations, we plan to conduct future studies with a larger WSI dataset collected from multiple scanners, aiming to investigate the influence of scanner variability and develop a more robust classifier suitable for clinical application. Furthermore, due to the loss of original tissue blocks, we were unable to perform additional immunohistochemical (IHC) staining to validate the identity of certain cells or explore specific inflammatory phenotypes. Another limitation lies in the absence of ***BAP1*** mutation data in our WCH cohort, which prevented us from conducting analyses or predictions specifically related to ***BAP1*** mutational status, despite its well-established role as a key driver of aggressive tumor behavior and metastatic potential in uveal melanoma. These constraints highlight the need for future investigations that incorporate a more comprehensive dataset, including molecular data and preserved tissue samples, to further validate and expand upon our findings.

In conclusion, our study reveals a potential close relationship between the histopathological morphology of tumors and their underlying molecular composition. By analyzing UVM histopathology images, high-performance automated diagnosis, subtyping, and prediction may be achievable, offering significant potential to improve UVM patient diagnosis, prognosis, and therapeutic strategies. Although our findings demonstrate the promise of these models in aiding clinical decision-making, further validation and integration into clinical workflows are required before they can directly guide individualized treatment plans and improve patient outcomes.

## Data Availability

The datasets presented in this article are not readily available due to restrictions from the West China Hospital data privacy policy. Requests to access the datasets should be directed to the corresponding author, and will be made available on reasonable request.

## Supplementary material

Supplementary Figure 1 The performance of the Inception-V3 deep learning model. **(A)** ROC curves. **(B)** Precision-recall curves. **(C)** Confusion matrices for the train dataset. **(D)** Confusion matrices for the validation dataset. **(E)** The specific metrics and detailed performance results of the model, including accuracy, precision, recall, F1-score, and AUC values.
