# Supplementary figures and images for: Integrating pathomics and deep learning for subtyping uveal melanoma: identifying high-risk immune infiltration profiles
Source: Front Immunol. 2025 Jul 9;16:1585097. doi: 10.3389/fimmu.2025.1585097 (PMC12283581; doi:10.3389/fimmu.2025.1585097)

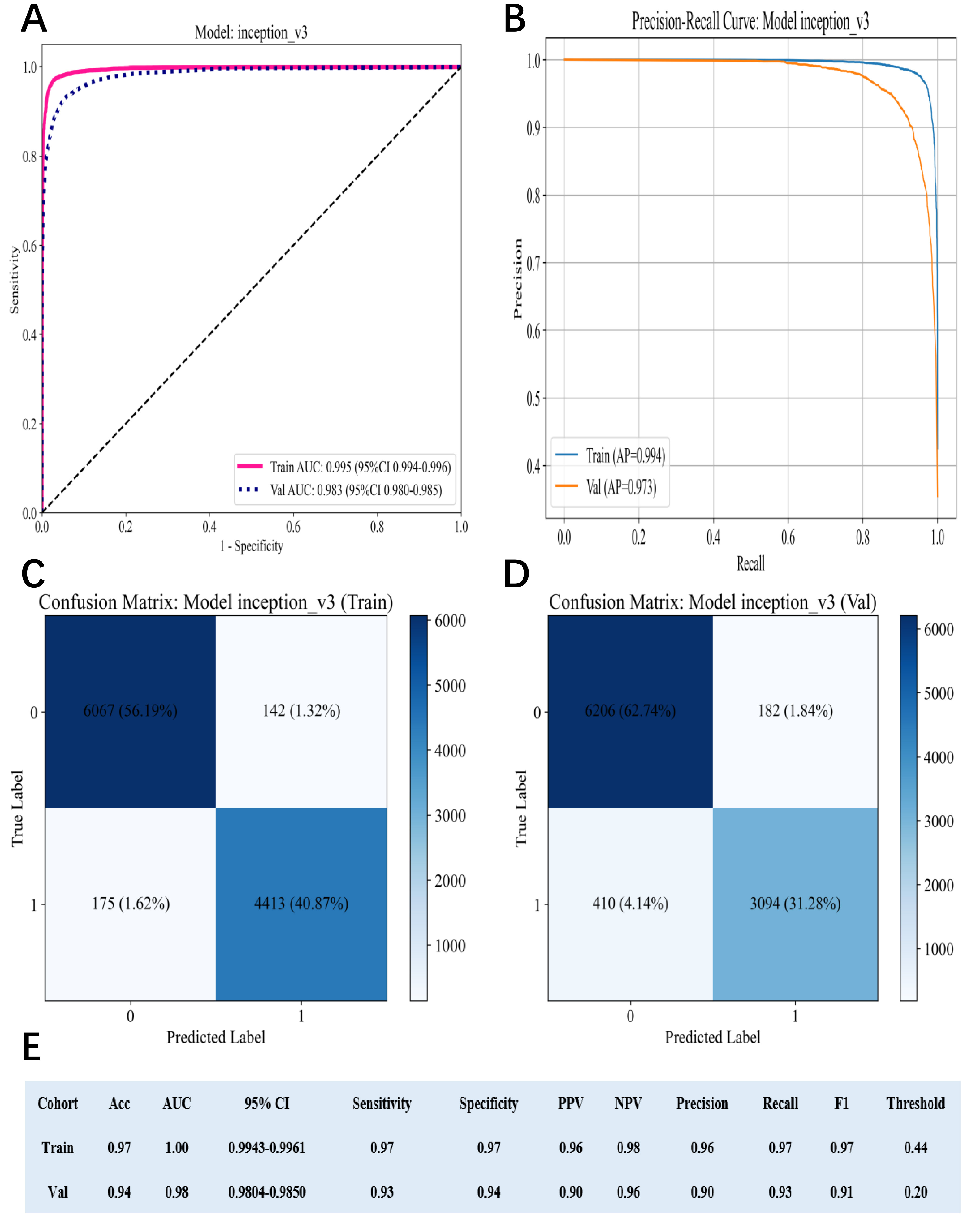

Supplement: Supplementary Figure 1 — The performance of the Inception-V3 deep learning model. (A) ROC curves. (B) Precision-recall curves. (C) Confusion matrices for the train dataset. (D) Confusion matrices for the validation dataset. (E) The specific metrics and detailed performance results of the model, including accuracy, precision, recall, F1-score, and AUC values. [file Image1.jpeg]
